# Genomic characterization of grapevine virus J, a novel virus identified in grapevine

**DOI:** 10.1007/s00705-018-3793-y

**Published:** 2018-03-07

**Authors:** Alfredo Diaz-Lara, Deborah Golino, Maher Al Rwahnih

**Affiliations:** Department of Plant Pathology, University of California, Davis, Davis, CA 95616 USA

## Abstract

This paper describes the nucleotide sequence and genome organization of a novel RNA virus detected in grapevine (*Vitis vinifera*) cultivar ‘Kizil Sapak’ by high-throughput sequencing (HTS) and tentatively named “grapevine virus J” (GVJ). The full genome of GVJ is 7,390 nucleotides in length, which comprises five open reading frames (ORFs), including a 20K ORF (ORF 2) between the replicase (ORF 1) and the movement protein (ORF 3) genes. According to the level of sequence homology and phylogenetics, GVJ is proposed as a new member of the genus *Vitivirus* (subfamily *Trivirinae*; family *Betaflexiviridae*), with the closest characterized virus being grapevine virus D (GVD).

The grapevine (*Vitis vinifera*) is considered one of the oldest crops in the history of humanity [1] and is exposed to different pests and pathogens. These pathogens include the vitiviruses (genus *Vitivirus*, subfamily *Trivirinae*, family *Betaflexiviridae*), whose type species is *Grapevine virus A* (GVA), which is associated with the etiology of rugose wood disease in grapevine [2]. Members of the genus *Vitivirus* have a positive-sense single-stranded (ss)RNA genome with five open reading frames (ORFs), including a distinctive 20K ORF between the ORF1 (replicase) and the ORF3 (movement protein) [3, 4]. To date, nine viruses are formally classified as vitiviruses (https://talk.ictvonline.org/), five of which are reported in grapevine: GVA, grapevine virus B, grapevine virus D (GVD), grapevine virus E and grapevine virus F (GVF) [2]. Four recently discovered viruses, grapevine virus G (GenBank: MF405923) [5], grapevine virus H (GenBank: MF521889) [6], grapevine virus I (GenBank: MF927925) (A. G. Blouin, personal communication), and grapevine virus K (GVK; GenBank: MF072319) [7], are proposed as members of the *Vitivirus* genus. Nevertheless, the taxonomy of GVK should be further investigated after a phylogenetic study indicated that this virus is a variant of GVD [5]. During the characterization of a new selection of white grape, *V. vinifera* cv. ‘Kizil Sapak’ (KS) from Turkmenistan, a novel vitivirus was detected and tentatively named “grapevine virus J” (GVJ). A reverse transcription polymerase chain reaction (RT-PCR) assay was developed and used to confirm the presence of the new virus in the source plant.

In 2014, the quarantine selection KS was received for inclusion in the Foundation Plant Services (FPS, UC-Davis, CA) collection. The vine was grown in a screenhouse and assayed for known grapevine viruses as described by Al Rwahnih et al. [8] and subjected to high-throughput sequencing (HTS) analysis as part of the routine testing procedure. Briefly, total nucleic acid extracts from leaf petioles were used as template for cDNA library construction [9]. The cDNA library was sequenced using the Illumina NextSeq 500 platform at the UC-Davis Genome Center and yielded approximately 32 million raw HTS reads, which were filtered and trimmed using CLC Genomics Workbench (QIAGEN). Contiguous consensus sequences (contigs) were generated from the cleaned HTS reads using the CLC *de novo* assembler and compared against the NCBI database of viruses using tBLASTx (https://blast.ncbi.nlm.nih.gov/Blast.cgi). The previous analysis revealed nine contigs that ranged in size between 4,743 to 7,375 nucleotides (nt) and showed a distant relationship (average identity: 52%) to several viruses belonging to the genus *Vitivirus*. Consequently, the longest contig and near-complete genome sequence (7,375 nt) of the putative virus was extended using a FirstChoice RLM-RACE kit (ThermoFisher Scientific) to obtain the 5’ and 3’ ends of the genome. The complete genome sequence (including a 12-nt polyA tail) of the new vitivirus, for which the name “grapevine virus J” (GVJ) is proposed, was 7,390 nt long (GenBank: MG637048; Fig. 1). The full-length virus genome sequence was compared with sequences in the NCBI database using BLASTn; the two best hits corresponded to GVD (75% identity; 12% query coverage) and the recently sequenced GVK (68% identity; 78% query coverage).

**Fig. 1:**
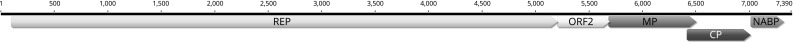
Genome organization of grapevine virus J (GVJ). The genome contains 5 predicted open reading frames (ORFs). REP, replicase; MP, movement protein; CP, coat protein; NABP, nucleic acid binding protein

ORFs were identified using ORF Finder (https://www.ncbi.nlm.nih.gov/orffinder/), and these coding regions were then analyzed independently using the SmartBLAST algorithm and the NCBI Conserved Domain Search Tool (https://www.ncbi.nlm.nih.gov/Structure/cdd/wrpsb.cgi). Finally, Protein Information Resource (http://pir.georgetown.edu/pirwww/search/comp_mw.shtml) was employed to calculate the molecular mass of each of the predicted proteins. The genome of GVJ was found to contain five putative ORFs and 5’ and 3’ untranslated regions of 95 and 69 nt, respectively. ORF1 encoded a polypeptide of 1,702 amino acids (aa) (194.6 kDa), was similar (52% aa identity and 99% query coverage) to the replicase gene of GVA, and contained the following domains: a viral methyltransferase domain (G_62_-K_340_) found in a wide range of ssRNA viruses; an alkylation B (AlkB) domain (H_620_-R_741_); a viral RNA helicase domain (G_931_-A_1147_) belonging to the superfamily 1; and an RNA-dependent RNA polymerase domain (D_1360_-W_1597_). A 17.7-kDa protein of unknown function was encoded by the ORF2 (164 aa). ORF3 (273 aa; 31.1 kDa) showed 52% aa sequence identity (95% query coverage) to the movement protein of GVA. A predicted coat protein (CP) with a molecular mass of 21.7 kDa was identified from ORF4 (197 aa), based on 86% aa sequence identity (81% query coverage) to the CP of GVD. Finally, the 103 aa (11.8 kDa) encoded by ORF5 showed similarity to a putative RNA-binding protein present in vitiviruses.

The phylogenetic analysis of GVJ was performed in MEGA v7.0.26 [10] using the Poisson aa substitution model and confirmed with 1,000 bootstrap pseudoreplicates. A neighbor-joining tree was obtained based on the aa sequence alignment of the replicase and CP genes of different members of the genus *Vitivirus* (viruses and the GenBank accession numbers are listed in Fig. 2). Grapevine Pinot gris virus (genus *Trichovirus*) was included as an outgroup. In the phylogenetic tree, GVJ clustered with members of the genus *Vitivirus* (Fig. 2), with GVD and GVK as sister taxa in both trees (replicase and CP).

**Fig. 2:**
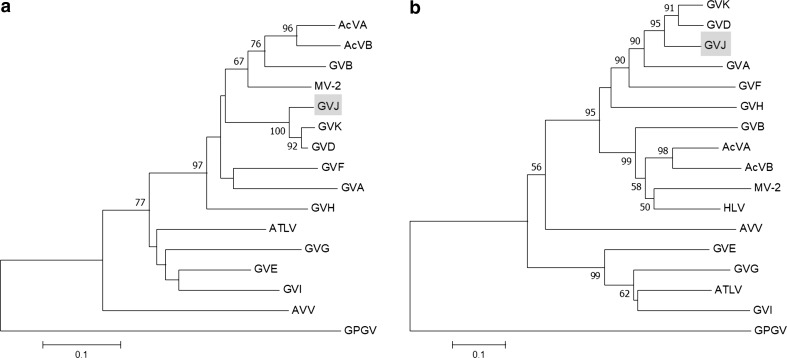
Phylogenetic inference of grapevine virus J (GVJ) in relation to members of the genus *Vitivirus*. Sequences of the following viruses were included in the analysis: actinidia virus A (AcVA, JN427014), actinidia virus B (AcVB, NC_016404), mint virus 2 (MV-2, AY913795), grapevine virus B (GVB, NC_003602), grapevine virus H (GVH, MF521889), grapevine virus F (GVF, NC_018458), grapevine virus A (GVA, NC_003604), grapevine virus K (GVK, NC_035202), arracacha virus V (AVV, NC_034264), grapevine virus E (GVE, NC_011106), agave tequilana leaf virus (ATLV, NC_034833), grapevine virus D (GVD, KX828708, Y15892), grapevine virus G (GVG, MF405923), grapevine virus I (GVI, MF927925), heracleum latent virus (HLV, X79270), and grapevine Pinot gris virus (GPGV, NC_015782). Neighbor-joining trees were constructed based on the amino acid sequences of the replicase (a) and coat protein (b) using the Poisson model of substitution. The horizontal branch length is proportional to the genetic distance; the scale bars represent changes per site. Bootstrap values less than 50% are not shown

Primers were designed to confirm the prevalence of GVJ in the original KS plant via one-step RT-PCR. The oligonucleotides GVJ-CP-F (5’-GGGTATGACCAGGGCATGTATC-3’) and GVJ-CP-R (5’-CTCTTGCAATGTGAGTTTGCTCAG-3’), which corresponds to sites that flank a conserved region of the viral CP gene, were used to generate a 396-bp amplicon. The RT-PCR reaction was performed using SuperScript II Reverse Transcriptase (Life Technologies) and GoTaq (Promega), and the thermocycling program consisted of 30 min at 52°C, 35 cycles of 30 s at 94°C, 45 s at 55°C, 1 min at 72°C, and a final elongation step of 5 min at 72°C. Subsequently, the KS selection tested positive for GVJ, and the PCR product was sequenced directly to confirm its identity.

Here, a new virus, grapevine virus J (GVJ) was identified in an asymptomatic KS grapevine by HTS. Based on its genomic arrangement, sequence similarity to other viruses and phylogenetic relationships, we propose that the identified virus should be placed within the genus *Vitivirus*, subfamily *Trivirinae*, family *Betaflexiviridae*. Considering the current species demarcation criteria for members of the genus *Vitivirus* [11], GVJ can be considered a member of a new species due to the low level of aa sequence identity (less than 80%) of its replicase gene to those of other vitiviruses.

Further work is needed to identify the mechanisms by which GVJ is transmitted. Field surveys and biological studies are underway to determine the prevalence of GVJ in commercial vineyards and to assess its effect on vine performances. In addition, the incidence of GVJ in other selections introduced to FPS and the USDA National Clonal Germplasm Repository in Winters, CA, will be determined using the newly developed RT-PCR assay.
